# Overview of the Characteristics of Micro- and Nano-Structured Surface Plasmon Resonance Sensors

**DOI:** 10.3390/s110201565

**Published:** 2011-01-27

**Authors:** Sookyoung Roh, Taerin Chung, Byoungho Lee

**Affiliations:** National Creative Research Center for Active Plasmonics Application Systems, Inter-University Semiconductor Research Center and School of Electrical Engineering, Seoul National University, Gwanak-Gu Gwanakro 599, Seoul 151-744, Korea; E-Mails: sookyoungroh@gmail.com (S.R.); taerinc@gmail.com (T.C.)

**Keywords:** surface plasmon resonance, nanostructure, localized surface plasmon, sensor

## Abstract

The performance of bio-chemical sensing devices has been greatly improved by the development of surface plasmon resonance (SPR) based sensors. Advancements in micro- and nano-fabrication technologies have led to a variety of structures in SPR sensing systems being proposed. In this review, SPR sensors (from typical Kretschmann prism configurations to fiber sensor schemes) with micro- or nano-structures for local light field enhancement, extraordinary optical transmission, interference of surface plasmon waves, plasmonic cavities, *etc*. are discussed. We summarize and compare their performances and present guidelines for the design of SPR sensors.

## 1. Introduction

Surface plasmon resonance (SPR) has been heavily utilized in optical bio-sensing applications, since its first development in 1983 by Liedberg *et al*. [1]. The resonant spectral response of SPR to a variation in external refractive index, as the result of a bio-molecular interaction, plays a critical role in chemical and biological sensing technology. It offers distinguishing characteristics that are advantageous for use in sensitive and label-free biochemical assays.

Plasmonic sensor technologies have flourished due to the dramatic recent progress made in micro- and nano-fabrication technology [2–4]. Meanwhile, researchers are now attempting to develop novel devices capable of overcoming the limitations of conventional SPR based sensors. In order to improve the analytic figures of merit of a plasmonic sensor system, it is necessary to understand the basic mechanism and rules used in designing parameters in previous plasmonic sensor systems.

In this review, we discuss the fundamentals of plasmonic sensing and review SPR sensors based on the typical Kretschmann configuration but also other schemes with micro- or nano-structures for local light field enhancement, extraordinary optical transmission (EOT), and interference of surface plasmon waves with emphasis on the design of the SPR coupler with a sensor-chip or a sensing probe. We summarize and compare their performances, and present guidelines for the design of SPR sensors.

## 2. Propagating SPR Based Sensor

### 2.1. Surface Plasmon Resonance based Sensor: Principle

Surface plasmon polaritons (SPPs) are defined as electromagnetic waves coupled with charge oscillations of free electrons in a metal that propagate along the boundary between the metal and a dielectric medium. When SPP excitation is optically induced, it is referred to as SPR.

**Figure 1.**
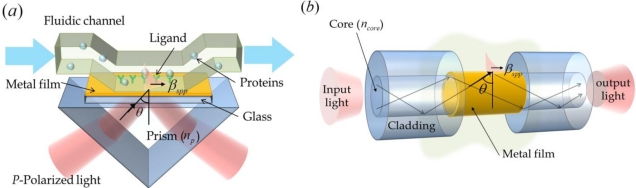

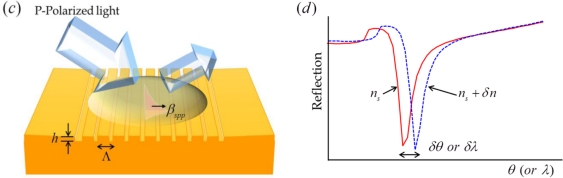
Basic schemes for SPR sensors with (**a**) Kretschmann configuration based coupling; (**b**) waveguide based coupling; (**c**) and grating coupling; (**d**) Reflection ratio of light due to SPR with angular modulation or wavelength modulation.

There are several fundamental methods available for exciting SPR, including prism coupling, waveguide coupling, and grating coupling methods as shown in Figure 1. The most conventional approach is the Kretschmann method, which employs a prism coupler with a thin metal film, as shown in Figure 1(a).

When TM-polarized (*p*-polarized) light is illuminated on the bottom side of a thin metal film through the prism, the resulting evanescently decaying field penetrates into the metal layer and reaches the upper boundary between the metal and sensing medium. This process effectively excites SPPs at the thin metal film. When SPR occurs, the incident light is absorbed by the metal film. Therefore, a resonance dip is produced in the reflection spectrum. The resonance condition is described as [5]:

(1) $$\frac{2\pi }{\lambda }n_{p}\;\text{sin}\;\theta =\beta_{\mathrm{ev}}=\text{Re}\;\left(\beta_{\mathrm{spp}}\right),$$

where *n_p_* is the refractive index of the dielectric prism, *λ* the wavelength in free space, and *θ* the incident angle of the illuminating light. To achieve a measurable resonance, the propagation constant *β_ev_* of the evanescent field induced from the TM incident light should correspond to the real part of complex *β_spp_*, which is the excited SPP propagation constant. The effective *β_spp_* at a thin metal film is somewhat different from the value at the interface between the bulk metal and the dielectric, *β*_*spp0*_ (*β*_*spp*_ = *β*_*spp0*_ + Δ*β*, $\beta_{\mathrm{spp}0}=\frac{\omega }{c}\sqrt{\frac{{ɛ}_{m}n_{s}^{2}}{{ɛ}_{m}+n_{s}^{2}}}$, Δ*β* accounts for the finite thickness of the metal film and the presence of the prism, *ɛ_m_* is the permittivity of the metal layer and *n_s_* is the refractive index of the sensing material) [5]. According to Equation (1), geometrical variables involving the thickness of the metal layer and the refractive index of a prism can be tuned to manage the SPR waveband or resonance angle in the Kretschmann configuration. In addition, the propagation constant of excited SPP *β_spp_* responds sensitively to the variation in the environmental refractive index. This property is typically adopted in order to improve the performance of SPR based sensors.

This resonance condition is also applicable for waveguide coupling based SPR sensors. Light injected into an optical fiber propagates into the core through total internal reflection and generates an evanescent field in the vicinity of the waveguide boundary, which induces SPR at the interface between the metal film and the sensing, as presented in Figure 1(b). A small portion of the sensing region in the fiber-optic sensor can be approximated as a 2D flat dielectric-metal-dielectric structure similar to a Kretschmann configuration. Meanwhile, the spectral response of fiber-optic SPR sensors is slightly different from the Kretschmann configuration. When an optical fiber is used as the sensor body, the spatial-frequency bandwidth of the angular spectrum of incident light at a point on the metal surface in the sensing region is quite wide and the control of incidence angle becomes difficult to implement. Because of these characteristics, many researchers have attempted to develop analytical procedures for estimating the performance of fiber-optic sensors [2].

The grating coupling method for SPP excitation is slightly different from the above described methods. SPPs can be produced by the direct illumination of a metal surface of a grating structure, as shown in Figure 1(c). To attain SPR, primary conditions are required. The component of the wave vector in the plane parallel to the grating surface is altered by diffraction (*m*·2*π*/Λ). The propagation constant of the wave vector in the plane of grating must be the same as the propagation constant of the SPPs, as described in the equation below [5]:

(2) $$\frac{2\pi }{\lambda }n_{d}\;\text{sin}\;\theta +m\frac{2\pi }{\Lambda }=\pm \;\text{Re}\;\left(\beta_{\mathrm{spp}}\right);\;\beta_{\mathrm{spp}}=\beta_{\mathrm{spp}0}+\Delta \beta ,$$

where *m* is an integer representing the diffraction order, *n_d_* is the refractive index of the sensing material, and Λ is the grating period. Here, Δ*β* accounts for the change in the SPPs propagation constant due to the presence of the grating structure.

The optical system of an SPR based refractive index sensor consists of a light source, an SPR coupler with a sensor chip, and a light detector. Various coupling methods are used to design an SPR coupler. To model SPR couplers properly, the geometrical properties of various SPR sensors should be numerically analyzed by employing calculation methods such as the finite element method (FEM) [6], the finite-difference time-domain method (FDTD) [7], the rigorous coupled wave analysis method (RCWA) [8], and others.

The performance of these plasmonic sensors is evaluated based on certain characteristic variables such as sensitivity, resolution and signal to noise ratio (SNR). Sensitivity is the main parameter to be considered in the design of a sensor system. In spectral interrogation, the resonance angle *θ_res_* or the resonance wavelength *λ_res_* is determined by the refractive index of the sensing medium, as mentioned above. Thus, when the refractive index of the sensing medium is altered by *δn*, the resonant angle of incidence light *θ_res_* is changed by *δθ* or the resonant wavelength *λ_res_* is changed by *δλ* as shown in Figure 1(d). Under these conditions, the sensitivity is defined as [9]

(3) $$S=\frac{{\delta \theta }_{\mathrm{res}}}{{\delta n}_{s}}\;\;\;\text{or}\;\;\;\frac{{\delta \lambda }_{\mathrm{res}}}{{\delta n}_{s}}$$

The resolution, or detection limit (DL), is adjusted by the smallest variation in the environmental refractive index that can be detected by the sensor. This can be deduced by taking into account the noise *σ* in the transduction signal and the sensitivity (*DL = σ/S*). This parameter is vital in terms of evaluating the capability of a sensor. DL definitely influences the spectral resolution of the detector for acquiring the output signal and can be improved by reducing the noise level [10,11]. The sensor detection limit can be also improved by increasing the sensitivity. Hence, we survey previous designs of sensor-chips and sensing probes with regard to sensitivity for major estimation of sensing performance. In order to achieve superior sensitivity and to decrease the detection limit of the sensor, we also need the resonance curve in the spectrum of the output signal that represents a small bandwidth and deep depth.

In the sections below, we discuss the basic roles of geometrical parameters in conventional SPR based sensors and introduce various types of sensors and their main characteristics. Methods for improving sensing performance are then discussed.

### 2.2. Metal-Film Coupling Based Sensors

#### 2.2.1. Kretschmann Configuration Based Sensors

SPR based sensors can be classified by the coupling types used, *i.e*., metal-film coupling based sensors and grating coupling based sensors. Among the metal-film coupling based sensors, let us first consider the Kretschmann configuration based sensor in Figure 1(a). Conventional SPR biosensors employ the standard Kretschmann configuration for the SPR coupler. They generally produce a sensitivity of about 5 × 10^−7^ refractive index units (RIU) [12]. Huang *et al.* recently reported a high angular sensitivity of over 500 deg. RIU^−1^ [13]. This was obtained by employing an appropriate low-index prism and using the optimal thickness of the metal film and a sufficiently large resonant angle at an appropriate wavelength. Similarly, better sensitivity for refractive index sensing can be achieved in the case of the Kretschmann configuration. Although the system is bulky, it is easy to fabricate practical sensors due to its simple structure, and the sensing response can be easily obtained by theoretical calculations of electromagnetic reflection and transmission at the flat metal film [14].

To construct a sensor based on the Kretschmann configuration, the effect of geometrical parameters must be considered. The thickness of metallic layers is a physical parameter of sensor structures and can be adjusted to improve the reflection spectrum. In addition, the spectral response of a sensor system is dependent on the wavelength of illuminated light and the refractive index of the prism.

**Figure 2.**
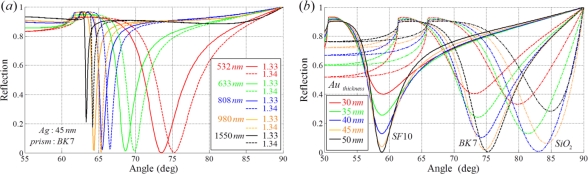
Reflection spectra of a Kretschmann configuration based SPR sensor **(a)** depending on the wavelength of the monochromatic light source with a BK7 prism and a 45 nm Ag metal film; and **(b)** response to various thickness of the Au metal layer with different materials used as the prism. In (b), a 633 nm monochromatic light source is assumed.

In SPR sensors with angular modulation of a monochromatic light, various wavelength sources can be utilized for illumination. When a longer wavelength light source is used for illumination, the angular spectral interrogation shows better resolution with a narrow bandwidth as shown in Figure 2(a). However, when the incident wavelength of the light source is increased, sensitivity is degraded. The increase in sensitivity at the short wavelength regime is associated with the effective index of a surface plasmon approaching the refractive index of the prism [9]. Based on the same principle, the sensitivity also depends on the material used in the construction of the prism. When the refractive index of the prism increases, the bandwidth of the reflection spectrum becomes narrow with a small resonance angle. However, sensitivity is degraded. In contrast, if the refractive index of the prism is smaller, the bandwidth of the resonance curve is broader [15].

To enhance SNR, metal thickness is an important factor. Figure 2(b) shows the reflection corresponding to the thickness of an Au layer with different prism materials. Although the optimal thickness of the metal layer is strongly influenced by the material of the substrate, approximate 50 nm is chosen as the optimal thickness for a conventional SPR sensing system. Various optimal conditions for Kretschmann configuration based sensors are presented in Appendix.

**Figure 3.**
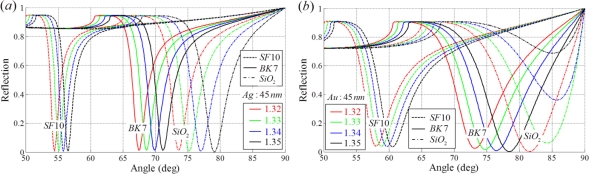
Reflection spectra of sensing response with different prism materials **(a)** with a 45 nm Ag metal film and **(b)** a 45 nm Au metal film (when a 633 nm light source for illumination).

To obtain a high sensitivity, a short wavelength source and a low refractive index prism covered with a thin Au film should be used. However, an appropriate choice is necessary because it can result in a broad bandwidth and a subsequent degradation of resolution. Figure 3 describes the sensing response of an SPR sensor on Ag and Au metal films for three prisms made of different materials at a fixed metal thickness. While silver presents the sharpest SPR resonance peak, gold has much better properties in terms of sensitivity and bandwidth. Gold has an excellent surface stability as well [2].

It is possible to determine the appropriate physical conditions in designing a sensor from reflection spectra. For example, the BK7 prism based SPR sensor, covered with a 50 nm thick Ag film, typically uses a 633 nm He-Ne laser as the light source.

To further enhance the sensing detection limit, various novel schemes have been investigated by using additional flat film layers such as dielectric over-layers [16] and a bimetallic layer [17]. Lahav *et al.* improved sensitivity by using a guided-wave surface-plasmon sensor configuration with an over-layer of a dielectric thin film having a high refractive index in 2008. Lee *et al.* demonstrated an SPR based sensor with an Ag-Au bimetallic layer inserted a ZnS-SiO_2_ waveguide layer. The use of a bimetallic layer has advantages in terms of the bandwidth of the SPR curve as well as in enhancing the local field at the Au surface interacting with analytes. Thus they obtained a 5.36 times higher sensing resolution than that of a conventional sensor (7.58 × 10^−6^ RIU).

**Figure 4.**
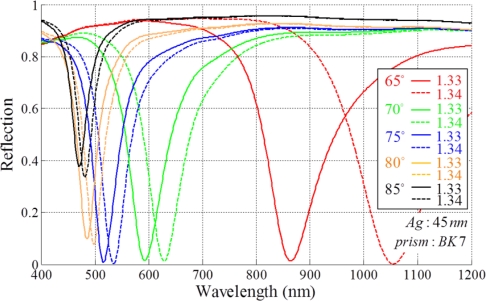
Reflection spectra of an SPR based sensor by varying the incident angle.

In SPR based sensors characterized by wavelength modulation, the sensing response is obtained by the coupling wavelength *λ_res_* at a fixed incidence angle and the response shows different patterns as the wavelength changes. As the resonance wavelength shifts to longer wavelengths, the sensitivity of the SPR sensors are improved [9]. Resonance wavelength is determined by the incidence angle of white light under conditions where other geometrical parameters are constant. If the incidence angle of the white light source is set to a small value, the resonance occurs with longer resonance wavelength and a high sensitivity. However, a broad bandwidth follows as shown in Figure 4. Homola *et al*. showed that optimization of this approach allows a sensitivity of 7,500 nm RIU^−1^ [18]. In addition, this property can be applicable for multichannel sensing systems [19–21].

In a Kretschmann configuration based SPR sensor, metallic nano-structures have been widely utilized to further improve the detection limit. Useful characteristics of metallic nano-structures include local field enhancement and metamaterial-like behavior. Sensitivity can be increased by increasing the surface area and local field enhancement occurs at nano-structures. In addition, grating structures are adopted for resonance wavelength or angle shifting by diffraction. Grating structures also used to induce a transmission through the metal film [22].

For example, Byun *et al.* designed an optimal Au nano-grating structure and, subsequently, investigated the effect of surface roughness on sensitivity [23]. They found that field localization on the nano-grating sidewalls resulted in a significant improvement in sensitivity. In their structure, the structural parameters for enhancing performance are the period of the array, the width, and height of the nano-wires. Those parameters can be effectively controlled by a numerical analysis using RCWA. In their configuration, for example, the nano-wire array had a period of 50 nm and a 5 nm thickness was employed. Such extremely shallow metallic grating structures with short periods have been shown to completely absorb incident light inside a wide angular interval [24].

In 2008, Chen *et al*. reported on a phase-based SPR sensor comprised of a gold nano-cylinder array with a three-dimensional nanostructure [25]. Their device consisted of 160 nm diameter and 400 nm height gold cylinders on a 47 nm flat Au film. By employing a heterodyne interference optical path, the resolution can reach 10^−7^ RIU. This value is doubled in comparison with a thin film SPR sensor for the detection of phase variation. Similarly, Kabashin *et al*. demonstrated a biosensor that utilized a plasmonic metamaterial that is capable of supporting a guided mode in a porous Au nanorod layer [26]. Their structures produced a significantly high sensitivity of more than 30,000 nm RIU^−1^ and a large probe depth (500 nm) in the infrared wavelength region. Their sensor exhibited the advantages of a plasmonic nano-structure, such as a tunable spectral response, a nano-scale bio-immobilization template and strong field localization inside the template matrix. These nano-scaled metallic structure arrays show optically metamaterial-like behavior when the period is several tens of nanometers (at least less than about 300 nm) [24]. The optical response of nanostructured metamaterials can be estimated using effective medium theory. The plasmonic resonance in a metamaterial is highly sensitive to the thickness of its metallic structure [27].

The configuration parameters for developing an SPR sensor suitable for a specific use can be obtained by applying a genetic algorithm (GA) [28]. The GA optimization method can be applied to various types of sensors. Therefore, we focus on investigations of structural parameters.

#### 2.2.2. Fiber-Optic SPR Sensors

Another type of metal-film coupling based SPR sensor is a waveguide based SPR sensor such as a fiber-optic SPR sensor. Optical fibers as SPR sensor bodies have been extensively studied in recent years. Fiber-optic SPR sensors have some advantages compared to other sensors, which include their capability of miniaturization, simplified optical design, and remote sensing using fibers and a high sensitivity due to SPR [2]. In a previous study, we determined the optimized structural parameters for a fiber-optic SPR sensor of a single mode fiber (SMF), in which a sensitivity of about 4000 nm RIU^−1^ was achieved when an Au metal-film (39 nm) was coated [28]. The principle of a waveguide based SPR sensor is similar to that of a Kretschmann configuration based sensor with wavelength modulation, as mentioned above. In this case, the transmission spectrum from the optical fiber comes from a few repeated reflections on the metal film in the sensing region. Each reflection corresponds to the reflection spectrum of a Kretschmann configuration with a low refractive index prism made of fused silica.

However, some difficulties are associated with analyzing and optimizing the performance of fiber-optic SPR sensors. Unlike Kretschmann configuration based SPR sensors, the wide spatial-frequency bandwidth leads to resonance curve broadening in the transmission spectrum. As a result, the resolution of the fiber-optic SPR sensor is deteriorated. Because of the limited incidence angles, the resonance curve is located in the visible region, when a flat Au or Ag metal layer is used [2]. Because of this, an analysis of a fiber-optic SPR sensor is more complicated than that for Kretschmann configuration based sensors. Moreover, since the fiber has a cylindrical configuration and the difference in the dimension between the optical fiber and metal layer is very large, a numerical analysis of a characteristic fiber-optic sensor without a low dimensional approximation involves a huge calculation burden. Hence in the numerical analysis method, such as the mode expansion and propagation method (MEP), matrix formalism for a multilayer system and the RCWA method, the planar structure approximation is usually used for their analytic approaches [2].

**Figure 5.**
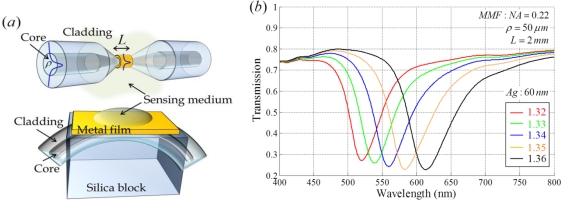
**(a)** Schemes for fiber-optic sensors with a tapered fiber and a D-shaped fiber (or polished fiber); **(b)** Transmission spectrum of a conventional fiber-optic SPR sensor with MMF (as shown in Figure 1(b)). The numerical aperture of the fiber is set to 0.22 and the core diameter is 50 μm. The length of the sensing region coated with a 60 nm Ag film is 2 mm.

For the modeling of a fiber-optic SPR sensor, it is necessary to carefully choose the type of fiber: an SMF or a multimode fiber (MMF). An SMF sensor generally exhibits a narrower resonance wavelength dip in the transmission spectrum than that of an MMF sensor. It presents better detection limit. When an MMF is employed for SPR sensor implementation, it is easy to detect output signals due to strong power intensity and the high SNR. However, an MMF based sensor is more sensitive to mechanical disturbances and launched conditions for the input light [29]. In the implementation of an actual fiber-optic SPR sensor system, MMF can be readily available. This is because optical fiber has a high propagation loss in the visible waveband and some types of MMFs have cladding made of a polymer that is easily removed. A small portion of the selected fiber is polished, etched or tapered and the metallic layer is deposited onto it, which has become common structure of a sensing probe as shown in Figure 5(a). Figure 5(b) presents the transmission spectrum of MMF when a portion of the cladding is symmetrically removed and an Ag film is coated on it. The transmission is numerically calculated using the three-dimensional ray-tracing method [30]. We can confirm that a fiber-optic SPR sensor shows a broader bandwidth and worse SNR than a Kretschmann configuration based sensor.

In a fiber-optic SPR sensor, structural modifications are actively applied to enhance sensing performance. Some approaches utilize distinct gratings, such as long period gratings [31] and tilted fiber Bragg gratings [32], to couple light from the core mode to the cladding modes and then provide the phase matching needed to achieve SPR on the surface of an optical fiber in the infrared region [31]. The use of a surface metallic Bragg grating has also been investigated [33]. These grating-employed SPR fiber sensors show somewhat degraded sensitivity in comparison with typical fiber-optic SPR sensors in the visible region. In these cases, the core mode is coupled to a hybrid cladding mode, which is weakly sensitive to a change in the effective index of the surface plasmon mode. In this case, most of the energy is associated with the guided mode and is only slightly weighted by plasmons [34]. However, the devices present sharp resonance dips and improved SNR. Metallic subwavelength nanostructures, in common with Kretschmann configuration based sensors, can be launched to fiber-optic sensor systems to induce the pure SPP mode and shift the resonance wavelength to the infrared region [28,35].

### 2.3. Grating Coupling Based Sensors

Metallic nano-structures can be transformed for grating coupling based SPR sensors. An SPP can also be excited by direct illumination of the gratings on a bulky metal surface.

Grating coupling based SPR sensors are typically less sensitive than metal-film coupling based sensors. Their resonance angle *θ_res_* and wavelength *λ_res_* are strongly dependent on the grating parameters involving period and fill factors, as previously mentioned in Equation (2). Hence, several theoretical and experimental studies have been carried out, in attempts to improve the performance of sensors, especially their sensitivity. The sensitivity of grating coupling based SPR sensors exhibits a minimum value that corresponds to the normal incidence (*λ =* Λ*n_spp_*) and increases with wavelength shift [9]. A grating coupling based sensor basically has geometrical parameters such as period, depth, and fill factor of gratings. Their optimization helps to improve sensing performance in both sensitivity and detection limit.

In 2006, Yoon *et al.* proposed nano-grating SPR sensors [36]. A refractive index sensitivity of more than 400 nm RIU^−1^ and relatively sharp resonance reflection peaks were obtained by vertical illumination. Their sensor, with a grating that has a feature size of less than 50 nm and a period of 500 nm, produced relatively sharp resonance reflection peaks. It would further enhance the sensing detection limits. In 2010, grating coupled SPR sensors using aluminum were reported by Hu *et al.* [37]. Compared with the Au-grating coupling based SPR sensor, their sensor demonstrated a better performance in SNR and detection limit. In their scheme, an ultrathin gold film was deposited on the grating surface in order to protect the Al layer from oxidation. Numerical simulations indicated that the angular sensitivity reached 187.2 deg. RIU^−1^ with a 900 nm light source and a grating period of 350 nm. To improve the sensitivity of grating-based systems in another way, Cai *et al*. realized sharp dips of the higher diffraction orders and proposed a double-dips method [38], using the separation of two sharp dips from different diffraction orders to improve the sensitivity of SPR sensors with a good linearity, 237 deg. RIU^−1^.

In addition to grating structures, diverse metallic nano-structures can be considered for SPR based sensor systems. For example, Liu *et al*. introduced a planar metamaterial structure in a plasmonic sensing system [39]. The structure consisted of an optically bright dipole antenna and a dark quadruple antenna, which are cut-out structures in the thin gold film. An outstanding reflectance peak was observed within a broad resonance curve by an electromagnetically induced transparency (EIT) like reflection. This metamaterial sensor was numerically analyzed by FDTD and experimentally demonstrated. As a result, it yielded a high sensitivity of 588 nm RIU^−1^ in the infrared region. Six geometrical parameters are controlled in this structure. Among the parameters, researchers considered lateral displacement as a key factor in determining the optimal spectrum. An in-depth analysis of all structural parameters could result in an improved performance.

Metallic nano-structures also show surface enhanced Raman scattering (SERS). It has been demonstrated that the Raman scattering of underlying biomolecules can be enhanced by localized SPR (LSPR) with metallic nano-structures. For example, spreading metallic nanoparticles on a metal film is an easy way to create roughness for LSPR [40]. This results in an enhancement of the light extinction on the surface. In LSPR based sensors with nanoparticles, the size and shape of the nanoparticles used are important parameters in terms of optimization.

## 3. Localized Surface Plasmon Resonance based Sensor

### 3.1. LSPR based Sensor: Principle

**Figure 6.**
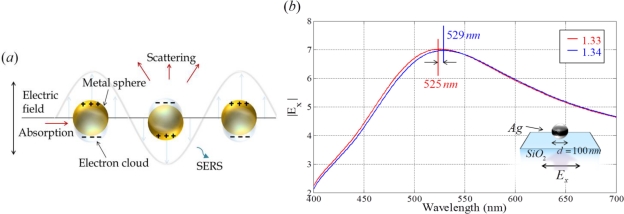
**(a)** Concept of localized surface plasmon resonance; and **(b)** spectrum of an enhanced amplitude of *E_x_* field from an Ag nanoparticle on a SiO_2_ substrate when the refractive index of the surrounding medium is changed from 1.33 to 1.34 RIU. The Ag nanoparticles have a diameter of 100 nm.

Recent significant advances in nano-fabrication and nanoparticle synthesis technology have made it possible to achieve the complicate patterning of metallic nano-structures. For nano-scaled metallic structures, it is possible to excite the localized oscillation of charges confined to the surface of nano-structures by light illumination, as shown in Figure 6 [41]. Conduction electrons in the nanoparticles oscillate collectively with a resonant frequency that is determined by their size, shape, composition and the refractive index of surrounding dielectrics [3,4]. This process is referred to as LSPR. Field enhancement of local electromagnetic fields on the surface of nanostructures arises by excitation of LSPR, and results in strong scattering and the absorption of light.

The resonance wavelength and electromagnetic field extinction (the sum of absorption and scattering cross sections) by LSPR are strongly dependent on the type of metal, size and shape of the nanostructures used. In the electrostatic dipole regime, the extinction *E*(*λ*) of a spherical metal-nanoparticle with size *a* is given as [42,43]

(4) $$E\left(\lambda \right)=\frac{24\pi^{2}{Na}^{3}{ɛ}_{d}^{3/2}}{\lambda \;\text{ln}(10)}\left(\frac{{ɛ}_{i}}{{\left({ɛ}_{r}+\chi {ɛ}_{d}\right)}^{2}+{ɛ}_{i}^{2}}\right)$$

where *λ* is the extinction wavelength and *χ* is a form factor that accounts for the aspect ratio of a spherical nanoparticle. *ɛ_d_* is the dielectric constant of the surrounding medium, and *ɛ_r_* and *ɛ_i_* are the real and imaginary parts of the dielectric function of metallic nanoparticles, respectively. In Equation (4), the particle is represented as *N* finite polarizable elements that can interact with the applied electric field individually. This approach for modeling the properties of a nanoparticle arises from the Mie theory. However, this analysis does not properly explain the optical behavior of larger metallic nanoparticles beyond the Rayleigh approximation [42]. Furthermore, in actual samples, nanoparticles are usually not spherical and many samples used in sensor systems contain an ensemble of nanoparticles that are supported on a substrate, which results in variation in the extinction spectrum.

LSPR by interacting with nano-structures leads to new optical responses. Near-field coupling or far-field dipolar interactions, depending on the spacing between adjacent nano-structures are induced [40,44]. For improved optical sensing, the LSPR and its resonance wavelength shift in a single nano-structure or interacting metallic nano-structures have been extensively exploited. Understanding LSPR properties and optimizing the design of nanoparticles are the main subjects of current plasmonic sensor research [41,42].

### 3.2. Plasmonic Nanoparticle Based Sensors

The sensitivity of optical sensors based on metallic nanoparticle arrays and a single nanoparticle have been substantially improved. Fundamentally, LSPR occurring at a nanoparticle is utilized to detect local changes in refractive index due to biological events in diverse sensing applications. In metallic nanoparticle sensors, nanoparticles are commonly immobilized on a glass substrate and exposed to aqueous solutions within fluidic channels. The wavelength scanning method is usually used for sensing the absorption, scattered or transmitted intensity from immobilized nanoparticles [40].

Using dark-field (optical scattering) microscopy, it is possible to directly observe nanoparticles by light absorption and scattering from LSPR [3,45]. Since a highly confined electromagnetic field is sensitive to a single molecule, smaller nanoparticles are advantageous for the detection of single molecules in bio-sensing. However, although a single nanoparticle enables sensitive chemical detection allowed by high spatial resolution, its size should be carefully determined to ensure that the signal intensity is sufficient for LSPR-shift assays. The absorption and scattering cross section of nanoparticles becomes comparable when the diameters are about 60 nm for Ag nanoparticles and 80 nm for gold nanoparticles [3,46].

For use in applications such as brightly colored spatial labels in immunoassays and cellular imaging, plasmonic nanoparticles also act as transducers that convert small changes in the local refractive index into spectral shifts in the intense nanoparticle extinction and scattering spectra. Molecular binding can be monitored in real time with a high sensitivity by using simple and inexpensive transmission spectrometry, which measures extinction. In general, a more sophisticated monitoring system is required to detect the scattering or absorption of incident light due to weak light intensity.

**Figure 7.**
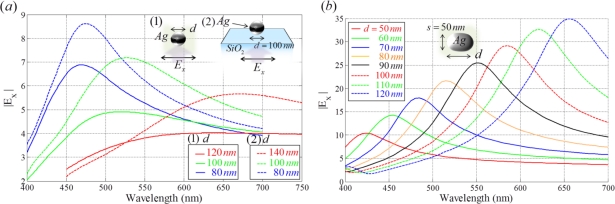
Spectra of enhanced *E_x_* field of **(a)** Ag nanoparticles (1) without or (2) with a substrate in aqueous solution (refractive index = 1.33) and **(b)** elliptical gold nanoparticles with various lengths of long axis. In (b), the short axis is fixed at 50 nm.

LSPR can be tuned during the fabrication process by controlling several parameters, such as the size and shape of nanoparticles and the dielectric constant of the substrate, using a variety of chemical synthesis methods and lithographic techniques [46–53]. Figure 7 illustrates the behaviors of an Ag nanoparticle with variation in particle size, substrate index and shape. Figure 7(a) shows the suitable diameter of a spherical nanoparticle in an aqueous solution at each red (650 nm), green, (530 nm), and blue (470 nm) wavelength resonance. As the diameter of a silver nanoparticle is increased, resonance of the silver nanoparticle is red-shifted. LSPR is enhanced more when the nanoparticles are supported on the substrate of a glass slide.

Increasing the aspect ratio (*d*/*s*) of nanoparticles and the refractive index of the solvent results in red-shifts in *λ_res_*, as shown in Figure 7(b). Extinction spectra of two nanoparticles with the same diameter have a larger cross-section than that for particles with different diameters. Pairs of nanoparticles that are separated by less than about 2.5 particle radii show plasmonic coupling and substantial spectral shifts. The spectral red-shift *δλ_res_* increases almost exponentially with a reduction in inter-particle spacing [4,53–55]. LSPR of a metallic nanoparticle-pair exhibits higher sensitivity to the refractive index of the environment as compared to a single nanoparticle. Tuning of plasmon resonance can also be achieved through strong electromagnetic coupling between the nanoparticle and the metal film. When the thickness of a thin silica spacer layer between the metal nanoparticle and the metal film is altered, the resonance frequency shifts [55].

Based on the above characteristics, a number of nanoparticle based sensors have been investigated. In 2004, Nath *et al.* fabricated a sensor from immobilized gold nanoparticles, which exhibited maximum sensitivity to the change of the bulk refractive index and the largest analytical volume with a 39 nm diameter [56]. Chen *et al.* distinguished the target from nonspecific binding in complex media by employing discrete gold nanoparticle dimers. Binding of the target DNA leads to a geometrical extension of the dimer, thereby yielding a spectral blue shift in the hybridized plasmon mode, as detected by single nanostructure scattering spectroscopy [57].

Au nanowires provide positional address and identification. By using this system, multiplexed sensing of target DNAs was possible in a quantitative manner. SERS employing nanoparticles has been considered for use in label-free multiplex DNA detection [58]. Kim *et al*. observed SERS spectra of brilliant cresyl blue (BCB), benzenethiol (BT), adenine and DNA from a controllable flower-like Au nanostructure array. The rougher Au nanostructure resulted in a higher SERS enhancement [59].

## 4. Other Types

### 4.1. EOT Based Sensors

**Figure 8.**
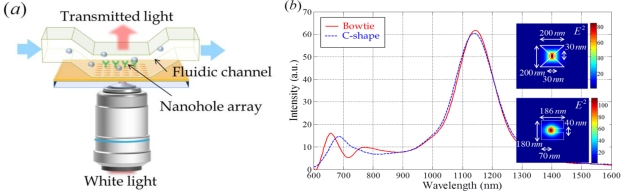
**(a)** Scheme for an EOT based sensor system with a periodic nanohole array in a metallic layer. **(b)** Transmission spectrum through single nanohole apertures — bowtie and C-shaped nano-apertures. The inset figures represent intensity distribution at a distance of 20 nm from each single nano-aperture.

Sensors based on EOT have also been investigated by many researchers. In 1998, Ebbesen *et al.* published a crucial experimental result on the transmission of light through nanohole arrays in thin noble metal films [60]. Their experiment indicated that transmitted light through nanohole arrays at certain wavelengths had a much higher intensity than estimated by the classical theory. The required condition for EOT through periodic nanohole arrays in a metal film coincides with the classical phase-matching condition for Bragg resonance. The resonance wavelength at normal incidence from periodic nanohole arrays with a square lattice can be estimated by the following Equation [61]:

(5) $$\lambda_{\mathrm{SP}}(i,j)=\frac{p\sqrt{\frac{{ɛ}_{m}n_{s}^{2}}{{ɛ}_{m}+n_{s}^{2}}}}{\sqrt{i^{2}+j^{2}}}$$

where *p* is the period of the array, and *i* and *j* are integers, denoting the scattering orders. Therefore, the transmission peak wavelength is determined by the refractive index of the surrounding dielectric *n_s_* and shift by the variation of *δn_s_*. With this condition, the phenomenon of EOT was investigated to develop a sensor system in which the configuration of the nanohole array is modified so as to achieve an enhancement in sensing performance, as shown in Figure 8.

An analytical expression of the spectral sensitivity for a two-dimensional nano-hole array was provided by Pang *et al.* in 2007 [62]. It is derived from an SPP dispersion relation. In their analysis, an EOT based sensor with an Au metal layer showed a sensitivity of 1000–1500 nm RIU^−1^ in infrared resonance conditions. Generally, EOT based sensors have a sensitivity of about 300 nm RIU^−1^ in the visible region (for example, a nanohole array with a thickness of 100 nm, a hole diameter of 200 nm, and a period of 500–600 nm) [61]. This sensitivity is smaller than the values for a commercial Kretschmann configuration based SPR sensor. However, the sensing area of the nanohole arrays is much smaller, which is the major merit of EOT based sensors. Hence, the magnitude of the wavelength shift originates from a smaller number of molecules in bio-sensing. Meanwhile, efforts to enhance the capabilities of the sensor continue to attract interest.

For enhancing sensing performance, a plasmonic sensing platform that exploits a nanohole array, which is perforated on a gold surface adhered on a fluoropolymer substrate, was described by Yang *et al*. in 2008 [63]. In their sensor, a fluorinated ethylene propylene copolymer (FEP) was chosen as the replica substrate because it is transparent in the visible region, and has a low refractive index (1.341 at *λ* = 590 nm) close to biological solutions. Using the low refractive index material makes the response of the sensor more sensitive to variations in the refractive index. In another approach for enhancing sensing capability, Artar *et al*. demonstrated an EOT effect through Fabry-Pérot cavities in multilayered plasmonic crystals. It is formed by coupling two physically separated metallic nanoholes and nanodisk array layers. It raises a strong electromagnetic field confinement in the dielectric region far from the metallic surfaces [64]. As a result, the cavity resonance was highly sensitive to changes in the refractive index.

EOT was also observed for single holes with contributions from LSPR. The transmission of light through a single hole depends strongly on its shape [65]. The enhanced localized electromagnetic field intensity increases the detection limit of a sensor. Various shapes of nano-holes and surface modifications have been actively investigated, in attempts to enhance the transmission, as shown in Figure 8(b). Various shapes of single nano-holes can be applied as periodic or aperiodic arrays. In periodic arrays, the linewidth of the transmission peak and its maximum intensity are influenced by each hole-shape [61].

### 4.2. Interferometry and Ring Cavity Based Sensors

In common with intensity detection due to absorption, phase variation caused by plasmonic resonance can be monitored using an interferometer or a resonator. In the case of interferometer based sensors, for example, an electro-optic heterodyne interferometer incorporating a Kretschmann configuration based sensor system is generally used to perform phase detection [25,66]. The performance of this interferometer based sensor is similar to or slightly less than that of the Kretschmann configuration based sensor and the system is bulky.

**Figure 9.**
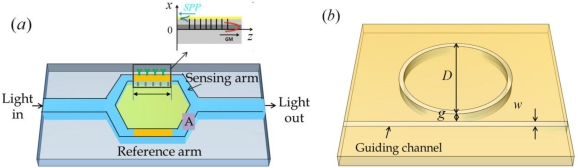
Schemes of SPR based sensors **(a)** with a Mach-Zehnder interferometer and **(b)** a disk resonator.

Typically, interferometry first separates the input beam into two beams with a beam splitter, exposes one of the separated beams to some type of external influence (e.g., refractive index changes in sensing medium), and then recombines the beams on another beam splitter. The power or the spatial shape of the combined beam can be measured to detect phase variation from the external influence [67]. Nenova *et al*. described a novel interferometer based an SPR sensor to detect a phase variation. This was accomplished by employing a simple integrated optical Mach-Zehnder interferometer [68]. SPP excitation is based on the resonance-coupling of the guided mode propagating in the waveguide layer with the contra- or co-propagating SPP supported by a metal layer. This is accomplished by means of an appropriately designed Bragg grating or long-period grating in the waveguide layer, as shown in Figure 9(a). This Mach-Zehnder interferometer can operate at commercialized telecommunication wavelengths. In the Mach-Zehnder interferometer, the sensing arm length and guiding waveband is determined. Another interferometry based sensor was proposed by Wu *et al.* in 2009. They described a refractive index sensor based on the interference of two surface-plasmon waves on both surfaces in a two-slit structure [69]. The sensor exhibited a linear response and a high sensitivity of 4547 nm RIU^−1^ using a gold film with a wavelength of 877.3 nm. Their result showed a high sensitivity but the detection signal was small.

**Figures 10.**
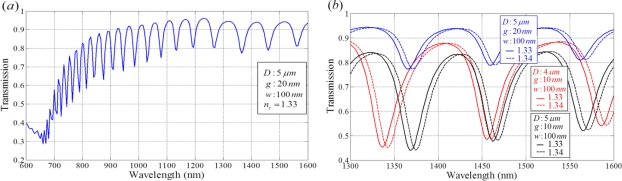
Transmission spectra of a plasmonic disk resonator. **(a)** Transmission spectrum for the ring cavity with 5 *μ*m diameter; **(b)** Transmission spectra of disk resonators with different geometric parameters, diameter (*D*) and gap (*g*) of the ring cavity, when the refractive index of the dielectric portion of the ring cavity is changed from 1.33 to 1.34 RIU. *w* is the waveguide width.

Another phase detection scheme involves the use of plasmonic ring resonators. The light propagates in a ring cavity with the form of whispering gallery modes or circulating waveguide modes. Each photon guided by total internal reflection circulates many times in the ring cavity. As a result, ring resonators provide a route for increasing sensitivity. One type of plasmonic ring resonator is a disk resonator, as shown in Figure 9(b) [70]. Using the schematics, we were able to obtain a transmission spectrum with narrow interference dips, as shown in Figure 10. The long path in the ring resonator increases the absorption of guiding light in the visible region in Figure 10(a). The valley wavelength in the infrared region is appropriate for use as the detection wavelength. When parameters of a ring radius of 5 *μ*m, a waveguide width of 100 nm and a 10 nm gap between the waveguide and ring structure were used, a sensitivity of 600 nm RIU^−1^ was achieved at a wavelength of approximately 1460 nm with a very narrow bandwidth as shown in Figure 10(b). The sensor signal is obtained from the interfered transmission spectrum of this disk resonator. In this case, the sensitivity improved as the wavelength red-shifts. The large radius of the disk resonator induces a narrow bandwidth and narrow spacing between wavelength locations that represent the minimum transmission values. However, the narrow spacing reduces the detection limit of the refractive index variation in the sensing medium (*δn_s_*). Based on resonance conditions in whispering gallery modes, the sensitivity of the resonance wavelength to changes in radius Δ*R* or refractive index Δ*n* is Δ*λ_res_* /*λ_res_* = Δ*R*/*R* + Δ*n*/*n* [71].

In addition, in the resonator, the material used in the resonator, channel width, and other parameters can also be adjusted for controlling the sensing response. Theoretical analyses of ring resonators and applications using various ring shapes have been actively investigated by researchers. In particular, Kim *et al.*, in 2007, demonstrated a novel biosensor based on the triangular resonator with TIR and ATR mirrors to achieve compactness and a high sensitivity [72].

Some types of sensor structures for detecting the phase variation are somewhat bulky, but the bandwidth of their output signal is narrow and wavelength selectivity in wide range of wavelength band including the infrared region. These are some advantages of a sensor with a Mach-Zehnder interferometer based or ring resonator based plasmonic sensor system.

In biosensor systems, a buffer layer containing specific bio-molecular recognition elements is coated on the surface of metal. This bio-layer recognizes and captures analytes present in a fluidic sample. This process leads to a local increase in the refractive index at the metal surface. The refractive index variation gives rise to an increase in the propagation constant of SPP (*β_spp_*), and this variation can then be accurately measured with the sensor [73]. However, there are some limitations because the surface plasmon wave is bound to the surface. The thickness of the buffer layer influences the sensing response and analyte capture should ideally occur near the metal surface. In particular, the response of an SPR based sensor varies in a complex manner and it is less sensitive when binding events occur at long distances from the metal surface. To overcome this limitation, the sensitivity of the SPR based sensor should be improved by means of appropriate design and novel detecting methods.

## 5. Conclusions

To conclude, an overview of design methods for surface plasmon based sensors is reviewed and categorized based on their optical structures. Table 1 presents the characteristics of some well-known SPR based sensors. Based on this review, it is possible to determine an appropriate method for achieving an ideal optical sensor. Due to the diverse variations of sensing objects, proper sensing structures should be selected for high sensitivity and biocompatibility. Prism and waveguide based structures originate from the reflection type based on the Kretschmann method. Nano-structure including nanoparticles, nanorods and nano-holes can show LSPR effect. By appropriate manipulation of structural parameters, the specific sensing schematics provided in this review can be implemented to fit a specific sensor purpose. However, many practical challenges such as instrument resolution and fabrication techniques remain, in terms of optimizing sensing performance.

## Figures and Tables

**Table 1.** Performance of SPR based sensors.

**Optical structure**	**Characteristics**	**RI range**	**Wavelength**	**Sensitivity**	**Ref.**
*Kretschmann configuration*					
Typical sensor	Au, Ag metal film	1.33–1.34	400–800 nm	100–300 deg./RIU	35
	Ag film, low index prism	1.328–1.332	1,310 nm	500 deg./RIU	13
	Au metal film	∼1.35	500–1,000 nm	7,500 nm/RIU, 10^−8^ RIU	18
Over layer	Au and Si, ZrO_2_ thin film	1.325–1.335	632.8 nm	50–230 deg./RIU	16
	Ag-Au bimetallic layer	1.33–1.34	632.8 nm	7.85 × 10^−6^ RIU	17
Nanostructured sensor	Au nano cylindrical array	1.33–1.335	632 nm	10-7/RIU	25
	Au nanorod metamaterial	∼1.33	1200–1300 nm	30,000 nm/RIU	26
	M-D mixed grating	1.33–1.36	633 nm	∼120 deg./RIU	22
Multichannel sensor	Dual channel, D over-layer	1.33–1.34	550–1150 nm	5 µg/mL α-DNA	20
	Angled polishing prism	1444–1.450	500–900 nm	2,710, 8,500 nm/RIU	19
*Fiber-Optic SPR sensors*					
Symmetrical cladding off	Au, Ag metal film	1.33–1.34	400–650 nm	2,000–4,500 nm/RIU	28
Grating	Cascaded LPG	1.33–1.39	∼1520 nm	−23.45 nm/RIU	
Nano-structured sensor	Au metallic grating	1.33–1.34	900–1,600 nm	4,000–9,800 nm/RIU	28
*Nano-structured-coupling*					
Grating-coupling	Au surface grating	1.33–1.34	∼600 nm	440 nm/RIU	
	Al-Au bimetallic layer	1.32–1.37	900 nm	187.2 deg./RIU	37
Metamaterial-like	Au nano-structured layer	1.332–1.372	∼150 THz (∼2,000 nm)	588 nm/RIU	39
*Nanoparticle based sensors*					
	Single or double-square periodic nanoparticle array	1.333–1.420	400–950 nm	200–350 nm/RIU	73
	Nanoparticle pair, disk pair	1–1.5	500–900 nm	172,434 nm/RIU	53
	Unperiodic array		300–700 nm	165 nm/RIU	74
Nano-structure	Gold nano-ring array	1–1.3	300–1,800 nm	637.3 nm/RIU	75
*EOT based sensors*					
	Square nanohole array	1.33–1.34	600–1,000 nm	300 nm/RIU	35
	Nanohole array		1,520–1,570 nm	1,110, 1,570 nm/RIU	62
	Fluoropolymer Substrates	1.33–1.37	∼600 nm	323 nm/RIU	27
*Interferometer*					
	Mach-Zehnder type	1.33	∼1,550 nm	250 nm/RIU	68
	Two slit interference	1.32–1.325	877.3 nm	4,547 nm/RIU	69
*Ring resonator*					
	Disk resonator	1.33–1.34	∼1,460 nm	600 nm/RIU	
	Triangular resonator		∼1,555 nm		72

## Appendix

Optimal conditions are numerically derived using theoretical calculations of electromagnetic reflection and the GA in Table A and Table B [2]. When the GA was applied, the objective function is designed as:

(A) $$F\left(P_{k}\right)=\sqrt{\sum_{j=1}^{3}{\left(a_{j}\left(\frac{y_{j}-y_{j\_\mathrm{ref}}}{y_{j\_\mathrm{ref}}}\right)\right)}^{2}}\;\text{with}\;\sum_{j=1}^{3}a_{j}^{2}=1$$

where *k* is the iteration number, *P_k_* is population. *y_1_*, *y_2_*, and *y_3_* are dependent parameters, each of which evaluates the bandwidth of the SPR dip, the SPR dip depth, and the sensitivity, respectively. *y_j_ref_* are the target values of respective parameters and *a_j_* is the weighting ratio of each evaluation factor, which is normalized as seen in equation (A). In the optimization, we assigned equal weight to the three elements.

In the tables, the term figure of merit (FOM) is defined by:

(B) $$\mathrm{FOM}=\frac{S}{\mathrm{BW}}\;\;\left({\mathrm{RIU}}^{-1}\right)$$

where *BW* and *S* denote the full-width at half-maximum of an SPR dip and the sensitivity, respectively. SPR sensing configurations exhibiting higher FOM provide higher resolution.

**Table A.** Optimal characteristics of a Kretschmann configuration-based sensor with an Ag layer.

**Wavelength (nm)**	**Substrate**	**Metal thickness (nm)**	**Resonance angle (deg.)**	**Sensitivity (deg./RIU)**	**FOM (RIU^−1^)**
532	SF10	47	57.4	80	26.7
	BK7	47	73.6	180	33.3
	SiO2	39	82.0	320	39.0
633	SF10	48	55.0	80	50.0
	BK7	50	68.6	120	50.0
	SiO2	49	75.2	200	62.5
808	SF10	48	53.4	80	133
	BK7	47	65.4	120	100
	SiO2	50	71.0	140	117
980	SF10	39	53.0	60	100
	BK7	44	64.4	100	125
	SiO2	48	69.4	140	175
1550	SF10	36	52.4	60	150
	BK7	36	63.4	100	167
	SiO2	33	68.4	120	120

**Table B.** Optimal characteristics of a Kretschmann configuration-based sensor with an Au layer.

**Wavelength (nm)**	**Substrate**	**Metal thickness (nm)**	**Resonance angle (deg.)**	**Sensitivity (deg./RIU)**	**FOM (RIU^−1^)**
532	SF10	38	63.4	100	4.58
	BK7	28	78.0	160	8.60
	SiO2	22	82.0	160	10.0
633	SF10	55	58.8	100	20.8
	BK7	51	75.2	200	23.8
	SiO2	39	82.6	280	27.5
808	SF10	45	53.6	80	80.0
	BK7	51	65.6	120	100
	SiO2	48	71.2	140	77.8
980	SF10	37	53.0	60	75.0
	BK7	41	64.4	100	100
	SiO2	43	69.4	140	117
1550	SF10	35	52.6	60	100
	BK7	39	63.6	100	125
	SiO2	39	68.6	120	120
